# Supplementary material for: A Review of Biologically Active Oxime Ethers
Source: Molecules. 2023 Jun 28;28(13):5041. doi: 10.3390/molecules28135041 (PMC10343442; doi:10.3390/molecules28135041)
Supplement: Supplementary file 1 [file molecules-28-05041-s001.zip › molecules-2454214-supplementary.pdf]

A review of biologically active oxime ethers

Tomasz Kosmalski [1*], Daria Kupczyk [2], Szymon Baumgart [1], Renata Paprocka [1] and Renata Studzińska [1*]

[1] Nicolaus Copernicus University in Toruń, Collegium Medicum in Bydgoszcz, Faculty of Pharmacy, Department of Organic Chemistry, 2 Jurasza Str., 85–089 Bydgoszcz, Poland; tkosm@cm.umk.pl, rstud@cm.umk.pl, sz.baumgart@cm.umk.pl, renata.bursa@cm.umk.pl
[2] Nicolaus Copernicus University in Toruń, Collegium Medicum in Bydgoszcz, Faculty of Medicine, Department of Medical Biology and Biochemistry, 24 Karłowicza Str., 85–092 Bydgoszcz, Poland; dariak@cm.umk.pl
* Correspondence: tkosm@cm.umk.pl, rstud@cm.umk.pl

Table S1. Minimal inhibitory concentrations (µg/mL) of the selected antifungal oxime ethers inhibiting growth of 80% and 90% of fungal strains (MIC_{80} and MIC_{90}, respectively).

	MIC_{80}			MIC_{90}	
	**11a**	**11b**	**11c**	**12a**	**12b**
C. albicans	0.004	0.016	0.016	2	16
C. parapsilosis	0.004	0.0625	0.0625	>128	>128
T. rubrum	0.0625	0.016	0.0625	-	-
A. niger	-	-	-	2	2
A. fumigatus	1	1	1	-	-
C. tropicalis	0.001	0.0625	-	-	-
C. krusei	0.0625	0.25	0.25	-	-
C. neoformans	0.016	0.001	0.004	32	16

Table S2. Minimal inhibitory concentrations (μg/mL) of the selected oxime ethers with antifungal activity.

MIC	**5a**	**5b**	**5c**	**6**	**7a**	**7b**	**7c**	**8a**	**8b**	**9**	**10a**	**10b**	**13a**	**13b**	**14**	**38**
C. albicans	4	2	1	2	4	16	16	0.25	0.5	>64	1	1	6.25	64	1	31.25
C. glabrata	0.5	1	0.06	4	-	-	-	-	-	-	-	-	-	-	-	-
C. parapsilosis	1	0.25	0.004	8	-	-	-	-	-	-	-	-	-	-	2	-
A. fumigatus	8	2	32	>128	-	-	-	-	-	-	-	-	-	-	-	-
A. flavus	32	8	32	>128	-	-	-	-	-	-	-	-	25	32	-	-
T. mentagrophytes	8	4	4	64	-	-	-	-	-	-	-	-	-	-	-	-
T. rubrum	8	2	2	64	-	-	-	-	-	-	-	-	-	-	-	-
S. cerevisiae	-	-	-	-	8	>64	32	0.5	2	-	-	-	-	-	-	-
A. niger	-	-	-	-	64	1	64	8	4	-	-	-	50	>128	-	-
M. gypseum	-	-	-	-	8	1	16	1	8	-	-	-	-	-	-	-
C. gatti	-	-	-	-	-	-	-	-	-	0.5	-	-	-	-	-	-
A. fumigatus	-	-	-	-	-	-	-	-	-	32	-	-	-	-	-	-
E. dermatitidis	-	-	-	-	-	-	-	-	-	64	-	-	-	-	-	-
C. krusei	-	-	-	-	-	-	-	-	-	-	-	-	-	-	1	-
Candida-51	-	-	-	-	-	-	-	-	-	-	-	-	6.25	32	-	-
Rhizopus	-	-	-	-	-	-	-	-	-	-	-	-	25	-	-	-

Table S3. Minimal inhibitory concentrations (μg/mL) of the selected oxime ethers with antibacterial activity.

	**30**	**31**	**32a**	**32b**	**36a**	**36b**	**37**	**38**	**40**	**41a**	**41b**	**42a**	**42b**	**42c**	**43**
S. aureus	0.19	0.781	32	32	62.5	62.5	1	7.81	6.25	3.125	12.5	0.5	0.5	0.5	-
MRSA	>100	-	-	-	-	-	-	-	-	-	-	-	-	-	-
S. epidermidis	0.19	-	-	-	-	-	-	-	-	-	-	-	-	-	-
B. subtilis	0.39	-	-	-	-	-	-	-	-	-	-	-	-	-	-
E. coli	0.39	0.391	32	32	125	125	-	125	12.5	50	25	>64	>64	>64	-
K. pneumoniae	0.19	1.563	-	-	-	-	>64	62.5	-	-	-	-	-	-	-
P. aeruginosa	25	>50	-	-	-	-	>64	-	6.25	>100	50	>64	>64	>64	-
S. pyogenes	-	-	32	32	-	-	-	-	-	-	-	-	-	-	-
S. typhimurium	-	-	32	64	-	-	-	-	-	-	-	-	-	-	-
A. baumannii	-	-	-	-	-	-	>64	-	-	-	-	-	-	-	-
M. tubercuosis	-	-	-	-	-	-	32	-	-	-	-	-	-	-	0.04
S. typhi	-	-	-	-	-	-	-	-	25	-	-	-	-	-	-
B. cereus	-	-	-	-	-	-	-	-	-	3.125	6.25	-	-	-	-
B. subtilis	-	-	-	-	-	-	-	15.62	-	-	-	-	-	-	-

MRSA: methicyllin-resistant *Staphylococcus aureus*

Table S4. Cytotoxicity (IC_{50}, µM) of the selected oxime ethers against selected human cancer cell lines

Line	**80a**	**80b**	**83**	**87**	**88**	**89a**	**89b**	**91a**	**91b**	**92a**	**92b**	**93**
K562	0.37	0.11	-	-	-	-	-	-	-	-	-	-
A549	-	-	-	2.0	-	40.2	47.4	-	-	-	-	-
HT-29	-	-	-	3.3	-	-	-	-	-	-	-	-
HeLa	-	-	8.43	22.6	5.63	-	-	6.6	7.5	-	-	-
MCF-7	-	-	11.06	>100	>30	-	-	6.6	7.5	-	-	0.075
MDA-MB-231	-	-	12.63	4.7	-	28.7	40.4	5.1	7.2	-	-	-
G-361	-	-	-	8.9	-	-	-	-	-	-	-	-
A431	-	-	-	-	13.25	-	-	-	-	-	-	-
A2780	-	-	-	-	25.05	-	-	-	-	-	-	-
PC-3	-	-	-	-	-	47.8	43.6	-	-	-	-	-
DU145	-	-	-	-	-	-	-	9.5	9.0	-	-	-
Ishikawa	-	-	-	-	-	-	-	6	12	-	-	-
SMMC-7721	-	-	-	-	-	-	-	-	-	0.64	0.63	-

Table S5. Selected mechanisms of action of the oxime ethers with the anticancer activity.

	Mechanism of action
**80a, 80b**	inhibition of tubulin polymerization
**87**	apoptosis
**88**	decrease in the G1 phase
**92a, 92b**	inhibition of migration and DNA replication, inducing irreversible apoptosis by regulating the expression level of apoptose-related proteins
**91b**	apoptosis, inhibition of migration and invasion, inhibition of cancer stem cell population, abrogation of EGF-induced proliferation, migration, and tyrosine kinase (TK) signaling in breast cancer cells
**93**	estrogen receptor agonist, antiproliferative activities via ER-independent mechanism
